# Expression of *E. coli* FimH Enhances Trafficking of an Orally Delivered *Lactobacillus acidophilus* Vaccine to Immune Inductive Sites via Antigen-Presenting Cells

**DOI:** 10.3390/vaccines11071162

**Published:** 2023-06-27

**Authors:** Allison C. Vilander, Kimberly Shelton, Alora LaVoy, Gregg A. Dean

**Affiliations:** Department of Microbiology, Immunology, and Pathology, College of Veterinary Medicine and Biomedical Sciences, Colorado State University, Fort Collins, CO 80523, USA

**Keywords:** mucosal vaccines, adjuvant, FimH, lactic acid bacteria, *Lactobacillus acidophilus*

## Abstract

The development of lactic acid bacteria as mucosal vaccine vectors requires the identification of robust mucosal adjuvants to increase vaccine effectiveness. The *E. coli* type I fimbriae adhesion protein FimH is of interest as a mucosal adjuvant as it targets microfold (M) cells enhancing vaccine uptake into Peyer’s patches and can activate the innate immune system via Toll-like receptor (TLR) 4 binding. Here, we displayed the N-terminal domain of FimH on the surface of a *Lactobacillus acidophilus* vaccine vector and evaluated its ability to increase uptake of *L. acidophilus* into Peyer’s patches and activate innate immune responses. FimH was robustly displayed on the *L. acidophilus* surface but did not increase uptake into the Peyer’s patches. FimH did increase trafficking of *L. acidophilus* to mesenteric lymph nodes by antigen-presenting cells including macrophages and dendritic cells. It also increased transcription of retinaldehyde dehydrogenase and decreased transcription of IL-21 in the Peyer’s patches and mesenteric lymph nodes. The N-terminal domain of FimH did not activate TLR4 in vitro, indicating that FimH may stimulate innate immune responses through a not-yet-identified mechanism. These results indicate that *E. coli* FimH alters the innate immune response to *L. acidophilus* and should be further studied as an adjuvant for lactic acid bacterial vaccine platforms.

## 1. Introduction

Mucosal vaccines offer advantages over parenteral delivery as they induce systemic as well as local immune responses which provide protection at the site where many pathogens cause infection. Oral vaccines must be gastric acid and bile resistant, able to access immune inductive sites such as the Peyer’s patches or mesenteric lymph nodes, and induce a robust immune response. The development of mucosal oral vaccines has been limited due to the challenges associated with vaccine delivery, and current licensed mucosal vaccines are restricted to attenuated live or killed pathogens [1]. Attenuated-live vaccines present a risk of reversion to virulence and thus cannot be used in immunosuppressed individuals. Additionally, they have been shown to have decreased effect in low- and middle-income countries [2]. The development of oral subunit or peptide vaccines has been constrained by low immunogenicity and the difficulty of targeting these vaccines to immune inductive sites. The discovery and evaluation of oral delivery systems and adjuvants to enhance immune responses is needed for the development of effective next-generation oral vaccines.

We have developed and evaluated the probiotic bacterium *Lactobacillus acidophilus* (LA) as an oral vaccine platform [3,4,5]. *L. acidophilus* is generally regarded as safe (GRAS) by the United States Food and Drug Administration, is gastric acid and bile resistant, and has endogenous mechanisms for activating the immune system. We have shown that recombinant LA (rLA) with a viral peptide embedded within the surface layer protein, SlpA, can induce systemic and mucosal immune responses [5]. Additionally, we have evaluated rLA cytokine secretion (proinflammatory cytokine IL-1β) and surface displayed *Salmonella typhimurium* flagellin (FliC, a Toll-like receptor (TLR) 5 ligand) as adjuvants [5]. In vivo, both adjuvant strategies increased antigen-specific immune responses following vaccination, indicating that one or more adjuvant strategies will likely be necessary for an effective rLA vaccine platform.

FimH is a type 1 fimbriae adhesion protein present on certain pathogenic *E. coli* and *Salmonella* spp. It is an important virulence factor for uropathogenic *E. coli* where it facilitates urogenital colonization via adhesion to Tamm–Horsfall proteins [6]. Microfold (M) cells are specialized cells that overlie Peyer’s patches and facilitate uptake into these immune inductive sites. Glycoprotein (GP) 2 is a specific marker of M cells and has a similar structure as the Tamm–Horsfall protein. It has been shown that *E. coli* and *Salmonella* spp. expressing FimH can bind to GP2 on M cells resulting in increased uptake of the bacteria into Peyer’s patches. Peyer’s patches are important for the induction of fecal IgA making FimH an attractive protein for use in an oral vaccine [7,8,9]. In addition to M cell targeting, FimH possesses other attractive immune-activating characteristics including binding to TLR4 and the macrophage CD48 receptor [10,11]. FimH is known to bind to M cells via the N-terminal domain, but the TLR4-activating domain of the FimH protein has not been definitively identified. The potential of FimH to perform as a vaccine adjuvant has been demonstrated via conjugation to epitopes of coxsackievirus B3, which resulted in increased Peyer’s patch uptake and immune response following oral delivery in mice [12]. Additionally, FimH has been identified as an immune stimulating adjuvant for cancer immunotherapy resulting in an increase in serum pro-inflammatory cytokines and dendritic cell maturation in mice in vivo [13].

Here, we evaluate the effect of rLA surface expression of the N-terminal domain of FimH on the uptake of our rLA vaccine into Peyer’s patches and innate immune response. Expression of the N-terminal (N-term) FimH by rLA was confirmed by Western blot and flow cytometry. We assessed the binding of rLA surface-expressing the N-term FimH to GP2, transcription of cytokines and enzymes in Peyer’s patches and mesenteric lymph nodes following oral delivery, and TLR4 activation in vivo and in vitro.

## 2. Materials and Methods

### 2.1. FimH Plasmid Construction and Lactobacillus acidophilus Transformation

Three N-term FimH expression plasmids were constructed by restriction digest and ligation into the plasmid pTRK1033 containing the Amub *L. acidophilus* surface anchor, a secretion signal, an origin of replication, and an erythromycin resistance gene [4]. The DNA sequence of the N-terminal domain (amino acids (AA) 22-181) of *E. coli* FimH (Uniprot Knowledgebase (UniProtKB) P08191) was optimized for *L. acidophilus* expression and ordered as a gene block from Integrated DNA Technologies (Coralville, IA, USA). N-term FimH sequences for plasmid construction were amplified by PCR with the following primers: 5′-FLAG-tag-FimH: 5′ FLAG-tag gene block with AV-09 and AV-10; 3′-FLAG-tag-FimH: 3′ FLAG-tag gene block with AV-09 and AV-10; and no-tag FimH: 3′ FLAG-tag gene block with AV-09 and AV-29. The PCR product was then further amplified with primers AV-09 and AV-30 (Supplemental Table S1). The plasmid (pTRK1033) was digested with the restriction enzymes NheI and KpnI, the digested plasmid and FimH PCR amplicon assembled via Gibson Assembly (New England Biolabs, Ipswich, MA, USA), and it was then transformed into competent DH5-ɑ *E. coli*. The resulting plasmids pD001, pD002, and pD003 were screened by PCR and then confirmed by sequencing (Table 1).

N-term FimH plasmids were transformed into LA strain GAD31 or GAD80, used as our model LA vaccines. GAD31 displays a peptide from the HIV-1 glycoprotein MPER (NEQELLELDKWASLWN), and GAD80 displays a 10-amino acid peptide (MASLIYRQLL) from the N-terminus of the rotavirus VP8 capsid protein within the surface layer protein A (SlpA). Construction of these strains was performed as described by Douglas et al. and Kajikawa et al. [5,14]. Plasmid transformation was performed based on a previously described protocol [14,15]. Briefly, GAD31 or GAD80 were washed with an isosmotic buffer (1000 mol/L sucrose and 2.5 mol/L MgCl_2_) and concentrated 100-fold. Concentrated rLA was mixed with approximately 500 ng of plasmid, transferred to a 2 mm-gap electroporation cuvette, and supplied with 2.5 kV, 400 Ω, and 25 µF electrical pulse. Transformants were selected on Luria agar containing 5 µg/mL erythromycin. The resulting rLA strains are listed in Table 1.

### 2.2. N-Term FimH Western Blot

Anti-FLAG-tag Western blot was used to confirm expression of N-term FimH for the GAD41 and GAD42 constructs. rLA was grown to exponential phase in deMan, Rogosa, and Sharpe broth (MRS) plus 5 µg/mL erythromycin and washed with phosphate-buffered saline (PBS) plus 1× protease inhibitor (ProteaseArrest, G Biosciences, St. Louis, MO, USA). Washed rLA were incubated overnight at 4 °C in PBS plus 1× protease inhibitor and mutanolysin (MilliporeSigma, Burlington, MA, USA). Washes were repeated and rLA homogenized via beating with 0.1 mm zirconia/silica beads (BioSpec, Bartlesville, OK, USA). The homogenized rLA was incubated with Benzonase (Millipore-Sigma, Burlington, MA, USA), mixed with Pierce Lane Marker Reducing Sample buffer (Thermo Scientific, Waltham, MA, USA), and proteins were separated by sodium dodecyl sulfate-polyacrylamide gel electrophoresis (SDS-PAGE) on a 4–20% Mini-PROTEAN TGX gel (Bio-Rad, Hercules, CA, USA) run with Precision Plus Protein WesternC Blotting Standard (Bio-Rad, Hercules, CA, USA). Proteins were transferred to a 0.45 µm polyvinylidene difluoride (PVDF) membrane using the Trans-Blot Turbo Transfer System (Bio-Rad, Hercules, CA, USA). The membrane was blocked with 1% non-fat dry milk in PBS + 0.05% TWEEN 20 (PBST) and protein labeled using 1 µg/mL rat anti-FLAG-tag primary (clone L5; Ebioscience, San Diego, CA, USA) followed by goat anti-rat IgG HRP (SeraCare, Milford, MA, USA) secondary antibodies. Chemiluminescence was detected using Bio-Rad Clarity enhanced chemiluminescence Western blotting substrate on a ChemiDoc XRS+ System (Hercules, CA, USA).

### 2.3. N-Term FimH rLA Surface Expression Flow Cytometry

N-term FimH surface expression was detected by flow cytometry. GAD31, GAD40, GAD80, and GAD83 were grown to exponential phase from overnight cultures and washed with PBS. rLA was resuspended in PBS and colony forming units (CFU) calculated based on the optical density at 600 nm. Then, 1 × 10^7^ CFU rLA were suspended in staining buffer (PBS + 1% fetal bovine serum (FBS)) plus mouse anti-FimH mAb824 (a gift from Dr. Svgeni V. Sorurenko, University of Washington, Seattle, WA) followed by goat anti-mouse IgG1 PE (P-21129; Invitrogen, Waltham, MA, USA). Flow cytometry was performed using a Gallios flow cytometer (Beckman Coulter, Indianapolis, IN, USA). Analysis was performed using FlowJo software (Ashland, OR, USA). Gates were set using rLA incubated with only the secondary antibody.

### 2.4. Murine GP2 Expression and Purification

A plasmid containing murine glycoprotein 2 (mGP2) was purchased from OriGene (Rockville, MD, USA). The mGP2 gene was amplified from the plasmid using primers AV-11 and AV-12 to add a 3′-His tag (Supplemental Table S1). The amplified fragment was cloned into the pND14 eukaryotic expression plasmid (a gift from Dr. Gary Rhodes, University of California, Davis, CA, USA) by restriction digest (AfeI and PmeI) and ligation and then transformed into competent NEB5α cells by heat-shock. Positive transformants were selected by antibiotic resistance and the pND14-mGP2-His plasmid confirmed by PCR and DNA sequencing.

pND14-mGP2-His plasmid was transfected into 293T cells using TransIT-LT1 transfection reagent (Mirus, Madison, WI, USA) based on the manufacturer’s recommended protocol. Then, 24 h after transfection, the medium was replaced with Opti-MEM reduced serum medium (Gibco, San Diego, CA, USA). The transformed cells were incubated for 48 h, the medium collected, and mGP2-His protein was purified by Capturem His-Tagged Purification Miniprep kit (Takara Bio USA, Mountain View, CA, USA). mGp2-His was confirmed by SDS-PAGE gel and Western blot with mouse anti-His tag HRP antibody (clone AD1.1.10, Bio-Rad, Hercules, CA, USA).

### 2.5. rLA FimH-Mouse GP2-Binding Assay

mGP2-His binding to FimH was confirmed by indirect ELISA using a modified protocol based on the method by Stoeker et al. [16]. GAD31, GAD40, GAD41, and GAD42 were grown in MRS plus 5 µg/mL erythromycin overnight. rLA were washed with PBS, and CFU was calculated by the optical density at 600 nm. rLA were suspended in PBS at a concentration of 1 × 10^8^ CFU/mL, and 100 µL of each rLA strain was added to 5 wells of a MaxiSorp 96-well plate (Thermo Scientific, Waltham, MA, USA). Plates were incubated at 45 °C overnight to evaporate PBS. Wells were then washed with PBST and blocked with 1% bovine serum albumin (BSA) in PBS for 2 h at room temperature. Wells were washed with PBST, and 100 µL purified mGP2-His diluted to 0.2 µg/mL in sample diluent (PBST + 1% BSA) was added to each well. The plates were incubated for 1 h at room temperature, washed with PBST, and GP2 binding detected by adding 100 µL of a mouse anti-His tag HRP antibody (clone AD1.1.10; Bio-Rad, Hercules, CA, USA) diluted to 2 µg/mL in PBST. After 1 h incubation at room temperature, the wells were washed with PBST and incubated for 15 min with 100 µL SureBlue Reserve TMB Peroxidase Substrate (SeraCare, Milford, MA, USA). The color development was stopped with 36.5 g/L HCl, and the absorbance was measured on a plate reader at 450 nm with 570 nm background subtraction. Absorbance levels are reported as the average of the five wells coated with the same strain of rLA.

### 2.6. Ethics Statement and Mouse Usage

All experiments involving the use of animals complied with all relevant regulations and with the guidelines and approval of Colorado State University’s Institutional Animal Care and Use Committee (IACUC 14-5332A). All animals were monitored daily for any signs of stress or illness and euthanized at the end of studies by either carbon dioxide inhalation or overdose of isoflurane followed by cervical dislocation.

All mice were wild-type female BALB/cJ obtained from Jackson Laboratories (Bar Harbor, ME, USA). Mice were maintained in specific pathogen-free conditions, housed socially (2–5 mice per cage) in commercially available individually ventilated cages, and provided with autoclaved bedding and enrichment. Animals were fed ad libitum commercially irradiated rodent chow (Teklad Global, Envigo, Indianapolis, IN, USA) and tap water filtered via reverse osmosis in autoclaved water bottles.

### 2.7. Intestinal Loop Peyer’s Patch rLA Uptake and Mesenteric Lymph Node Trafficking

GAD31 and GAD40 were grown at 37 °C overnight, diluted 1:10, and grown to exponential phase in MRS plus 5 µg/mL erythromycin. rLA were washed with endotoxin-free PBS, and CFUs were calculated based on the optical density at 600 nm. Twelve 8–12-week-old female BALB/cJ mice were held without food for 16 h and anesthetized via isoflurane inhalation. rLA were injected into an isolated segment of the small intestine based on the intestinal loop method described by Fukuda et al. [17]. Briefly, the abdomen of anesthetized mice was prepped via repeated scrubbing with 70% ethanol and the fur removed by clippers. The small intestine was isolated by encircling ligatures at the pyloric and ileo-cecal junction. Then, 1 × 10^9^ CFU rLA (GAD31 or GAD40) in 200 µL STI buffer (20 mg/mL soy trypsin inhibitor plus 8.5 mg/mL bicarbonate buffer) was delivered into the isolated small intestine. The abdomen was closed with skin clamps and the mice maintained under anesthesia for 1 h post-rLA delivery. Mice were euthanized via overdose of isoflurane followed by cervical dislocation. The small intestine and mesenteric lymph nodes were collected into RPMI 1640 (Corning 15-040-CV, Tewksbury, MA, USA) supplemented with 10 µg/mL glutamine and 10 mol/L HEPES (collection medium).

The small intestine was washed with cold PBS, religated with suture, the lumen infused with 1 mL cold 10% n-acetyl-L-cysteine pH 7.4 (Sigma-Aldrich, Darmstadt, HR, Germany), and incubated for 10 min on ice to digest intestinal mucus. The intestine was washed with cold PBS and Peyer’s patches collected by dissection into cold collection medium. Peyer’s patches and mesenteric lymph nodes were incubated for 30 min at 37 °C in RPMI supplemented with 10% FBS, 10 µg/mL glutamine, 10 mol/L HEPES, 1× MEM non-essential and essential amino acids, 1 mol/L sodium pyruvate, 0.055 mol/L 2-mercaptoethanol, and 10 µg/mL gentamycin to kill any remaining surface bacteria. Peyer’s patches and mesenteric lymph nodes were processed into a single-cell suspension by physical disruption, filtered, and live cells counted using a Cellometer Auto2000 (Nexcelom Bioscience, Lawrence, MA, USA). Cells were suspended in 1× RIPA buffer (Thermo Fisher Scientific, Waltham, MA, USA) at 5 × 10^6^ cells/mL and incubated for 5 min on ice to lyse mammalian cells. The cellular lysate was plated using an Eddy Jet spiral plater (Neutec Group Inc., Farmingdale, NY, USA) on MRS agar plus 5 µg/mL erythromycin to select for rLA. Plates were incubated under anaerobic conditions for 48 h and colonies counted using a flash and grow automatic colony counter (Cole-Parmer, Vernon Hills, IL, USA).

### 2.8. Mesenteric Lymph Node Antigen-Presenting Cell Flow Cytometry

GAD31 or GAD40 (5 × 10^9^ CFU) were suspended in 1 mL PBS plus 5 mol/L CellTrace Violet (Thermo Fisher Scientific, Waltham, MA, USA) and incubated on ice. Labeling was stopped with 10% FBS in PBS, and then rLA were diluted to 5 × 10^9^ CFU/mL in soy trypsin inhibitor bicarbonate buffer. CellTrace Violet labeling was confirmed by flow cytometry. Then, 10–12-week-old female BALB/cJ mice were fasted for 16 h and then orally dosed with 200 µL resuspended CellTrace Violet-labeled rLA. Subsequently, 1 h after oral delivery, mice were euthanized by CO_2_ asphyxiation followed by cervical dislocation and mesenteric lymph nodes collected into collection buffer. Mesenteric lymph nodes were processed into single-cell suspensions by physical disruption. Cells were blocked with anti-mouse CD16/32 (clone 93; BioLegend, San Diego, CA, USA) and labeled in staining buffer with a cocktail of 7-AAD viability staining solution (BioLegend, San Diego, CA, USA), 0.2 mg/mL PE anti-mouse CD64 (clone X54-5/7.1; BioLegend), 0.2 mg/mL PE/Cy7 anti-mouse I-A/I-E (clone M5/114.15.2; BioLegend), 0.5 mg/mL Alexa Fluor 647 anti-mouse CD103 (clone 2E7; BioLegend), 0.2 mg/mL APC/Cy7 anti-mouse/human CD11b (clone M1/170; BioLegend), 0.5 mg/mL biotin hamster anti-mouse CD11c (clone HL3; BD Pharmingen, San Jose, CA, USA), and 0.5 mg/mL FITC streptavidin (BioLegend). Flow cytometry was performed using a Gallios flow cytometer (Beckman Coulter, Indianapolis, IN, USA). Analysis was performed using FlowJo software (Ashland, OR, USA). After selecting for live cells, gates were set using fluorescence minus one (FMO) controls (Supplemental Figure S4A).

### 2.9. N-Term FimH TLR4 Activation Assay

HEK-Blue human TLR4 and HEK-Blue Null2 cell lines (InvivoGen, San Diego, CA, USA) were grown in cell culture treated flasks (Corning, Tewksbury, MA, USA) to confluency in DMEM media (15-013-CV; Corning) plus 10% heat-inactivated FBS, 10 µg/mL glutamine, 50 µg/mL penicillin/streptomycin, and 100 µg/mL Normocin (InvivoGen, San Diego, CA, USA) (cell culture media). After two passages, media was supplemented with selection antibiotics (TLR4: HEK-Blue Selection and Null2: Zeocin (InvivoGen, San Diego, CA, USA)). A total of 25,000 cells were plated on 96-well cell culture treated plates (Greiner Bio-One, Monroe, NC, USA) in cell culture media without antibiotics. Plates were maintained at 37 °C and 5% CO_2_ for 4 h prior to the addition of rLA.

GAD31 and GAD40 were grown at 37 °C overnight, diluted 1:10, and grown to exponential phase in MRS plus 5 µg/mL erythromycin. rLA were washed with endotoxin-free PBS, and CFUs were calculated based on the optical density at 600 nm. rLA were diluted in PBS, and 6.25 × 10^5^ and 1.25 × 10^6^ CFU rLA (correlating to multiplicity of infection (MOI) of 25 and 50) were added in duplicate to the HEK-Blue human TLR4 and HEK-Blue Null2 plated cells. Amounts of 100 ng and 10 ng of LPS were added as a positive control, and PBS was used as a negative control. rLA and cells were incubated at 37 °C and 5% CO_2_ overnight. An amount of 20 µL supernatant from each well was collected and added to 180 µL QUANTI-Blue Solution (InvivoGen, San Diego, CA, USA) and incubated at 37 °C for 4 h. Colorimetric change was detected by absorbance measured at 650 nm on a plate reader.

### 2.10. Peyer’s Patch and Mesenteric Lymph Node Quantitative Real-Time PCR Cytokine Evaluation

GAD80 and GAD83 were grown at 37 °C overnight, diluted 1:10, and grown to exponential phase in MRS plus 5 µg/mL erythromycin. rLA were washed with endotoxin-free PBS, and CFUs were calculated based on the optical density at 600 nm. Then, 10–12-week-old female BALB/cJ mice were orally dosed with 200 µL dosing buffer or rLA (GAD80 or GAD81) for a total of 1 × 10^9^ CFU in 600 nm 5 × 10^9^ CFU/mL in soy trypsin inhibitor bicarbonate buffer. Then, 24 h after delivery, mice were euthanized by CO_2_ asphyxiation followed by cervical dislocation, and Peyer’s patches and mesenteric lymph nodes were collected in collection media. Tissues were processed into single-cell suspensions. Peyer’s patches were mechanically disrupted using a gentleMACS Dissociator (Miltenyi Biotec, Auburn, CA, USA), and mesenteric lymph nodes were mechanically disrupted by mashing through a 100 µm cell strainer. All processed samples were filtered and live cells counted using a Cellometer Auto2000 (Nexcelom Bioscience, Lawrence, MA, USA). Between 1 × 10^6^ and 1 × 10^7^ live cells were washed once with cold PBS and then centrifuged, and the pellet was frozen at −80 °C. RNA was extracted using the Quick-RNA MiniPrep extraction kit (Zymo Research, Irvine, CA, USA) by the manufacturer’s protocol. RNA concentrations were determined with the Qubit RNA Broad-Range Assay Kit (Thermo Fisher Scientific, Waltham, MA, USA). RNA was diluted to 5 ng/µL and frozen at −20 °C until qRT-PCR assay.

The qRT-PCR assay was run in skirted white 96-well PCR plates (Bio-Rad, Hercules, CA, USA) using Luna Universal Probe One-Step RT-qPCR SuperMix (New England Biolabs, Ipswich, MA, USA). RNA extracted from mesenteric lymph nodes and Peyer’s patches was evaluated for expression of the cytokines and enzymes TGF-β, ALDH1A1 (retinaldehyde dehydrogenase 1), ALDH1A2 (retinaldehyde dehydrogenase 2), TNFSF13B (B cell activating factor (BAFF)), IL-21, IL-6, and the housekeeping genes HPRT and B2M using primer pairs and probes designed by Integrated DNA Technologies (Coralville, IA, USA). Primer pairs and probes are reported in Supplemental Table S2. Primer efficiencies ranged from 93 to 107%. All samples were randomized and run in duplicate with a pooled inter-plate control sample. Additionally, the SPUD assay was performed on each sample to screen for the presence of inhibitors [18]. Plates were run on Bio-Rad CFX96 Touch Real-Time PCR Detection System, and results were analyzed using Bio-Rad CFX Maestro Software.

Cytokines were normalized to averages of the reference genes HPRT and B2M. Fold change in cytokine expression was determined by calculating the Ct compared to the dosing buffer only mouse group and then transformed using Log_2_.

### 2.11. Statistics

Significant differences between groups were determined by a Kruskal–Wallis one-way ANOVA using GraphPad Prism 8.1.0 for windows (GraphPad software, San Diego, CA, USA).

## 3. Results

### 3.1. The N-Terminal Domain of E. coli FimH Can Be Detected on the Surface of Lactobacillus acidophilus by Flow Cytometry and Binds Murine GP2 In Vitro

**Figure 1:**
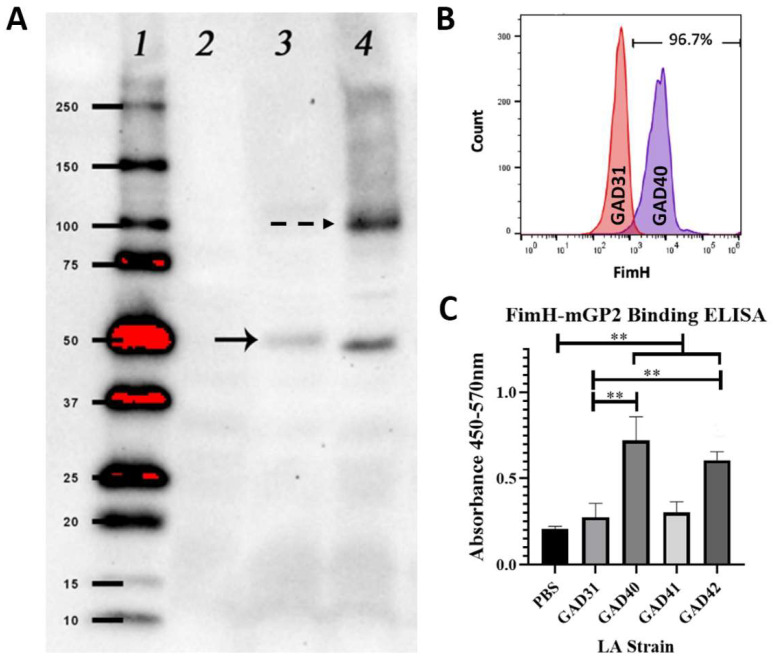
**GAD40 N-term FimH expression and in vitro N-term FimH rLA mGP2 binding.** (**A**) N-term FimH expression was confirmed by Western blot using an anti-FLAG antibody to detect FimH-FLAG on extractions of rLA surface layer proteins. A band (solid arrow) correlating to the molecular weight 44 kDa was present for both GAD41 (lane 3) and GAD42 (lane 4) and not observed for GAD31 (lane 2). An additional band (dashed arrow), present for GAD42, corresponds to a N-term FimH dimer. (Uncropped blot: Supplemental Figure S2; densitometry readings: Supplemental Table S3) (**B**) N-term FimH surface expression for GAD40 was confirmed by flow cytometry using an antibody specific for the FimH ligand-binding domain. GAD31 was the negative control. (**C**) mGP2 binding to MaxiSorp plates coated overnight with GAD31, GAD40, GAD41, or GAD42 was measured by 450–570 nm absorbance. mGP2 binding was significantly higher for GAD40 and GAD42 than GAD31. ** *p* < 0.05 based on a Kruskal–Wallis one-way ANOVA. mGP2: mouse glycoprotein 2; LA: *Lactobacillus acidophilus*.

FimH is the ligand-binding domain of the *E. coli* Type I fimbriae and consists of 279 AA that form two domains: an N-terminal ligand-binding domain (AA 1-156) and a C-terminal domain (AA 160-279) that complexes with the fimbriae protein FimC [19,20]. Full-length FimH has been shown to have decreased solubility if not expressed as a FimH–FimC complex. The N-terminal domain is readily expressible and soluble while maintaining the biologically active GP2-binding domain. Based on the solubility constraints of using full-length FimH, the FimH N-terminal ligand-binding domain with and without a FLAG-tag was cloned into plasmid pTRK1033 [4]. The N-term FimH plasmids were transformed into our vaccine constructs GAD31 (creating GAD40 (N-term FimH), GAD41 (N-term FimH + 5′-FLAG-tag), and GAD42 (N-term FimH + 3′-FLAG-tag)) and GAD80 (creating GAD83 (N-term FimH)). The expression of N-term FimH was confirmed by anti-FLAG Western blot (GAD41 and GAD42) (Figure 1A), and the surface expression was confirmed by flow cytometry (GAD40 and GAD83) (GAD40: Figure 1B, GAD83: Supplemental Figure S1).

The ability of rLA N-term FimH to bind mGP2 was assessed by an in vitro ELISA binding assay using mGP2 expressed in 293T cells and purified by His-tag. mGP2 binding was significantly higher (*p* < 0.05) for GAD40 and GAD42 than GAD31 or PBS only, confirming that N-term FimH expressed by rLA retains its GP2 binding activity (Figure 1C). The GAD41 strain binding to mGP2 was not significantly higher than GAD31, suggesting that the 5′-FLAG-tag on this construct blocked mGP2 binding.

### 3.2. N-Term FimH Increases Trafficking of rLA to Mesenteric Lymph Nodes, Which Is Mediated by Macrophages and Dendritic Cells

**Figure 2:**
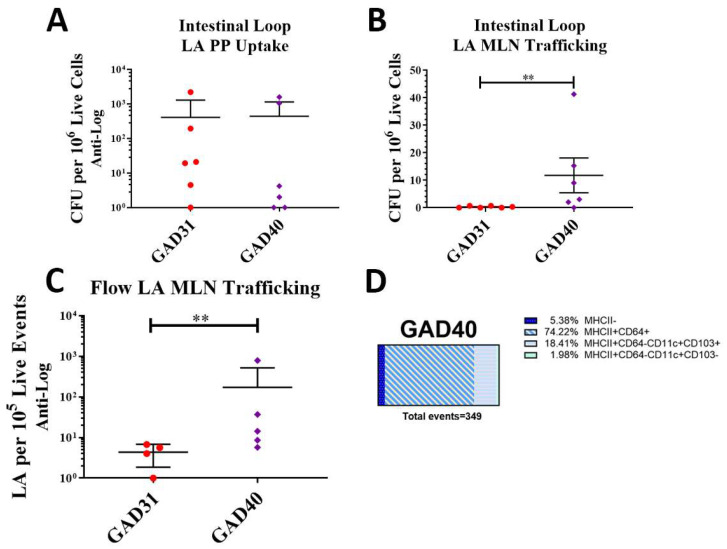
**rLA Peyer’s patch uptake and mesenteric lymph node trafficking.** Peyer’s patch rLA uptake and mesenteric lymph node rLA trafficking were assessed by intestinal loop (**A**,**B**). GAD31 or GAD40 were directly inoculated into an isolated section of small intestine in anesthetized BALB/cJ mice. Peyer’s patches and mesenteric lymph nodes were collected and processed into single-cell suspensions and plated on erythromycin-containing plates. There was no difference in Peyer’s patch rLA uptake between GAD31 and GAD40 (**B**), but there was a significant increase in GAD40 mesenteric lymph node trafficking compared to GAD31 (**A**). rLA mesenteric lymph node trafficking and antigen-presenting cell association following oral delivery of CellTrace Violet-labeled GAD31 and GAD40 (**C**,**D**). Significantly more CellTrace Violet-labeled GAD40 was detected in the mesenteric lymph nodes by flow cytometry than GAD31 (**C**). GAD40 was often associated with antigen-presenting cells (MHCII^+^) (**D**). ** *p* < 0.05 based on a Kruskal–Wallis one-way ANOVA. Flow gating and representative scatter plots of CellTrace Violet-labeled LA are shown in Supplemental Figure S1. Macrophages: MHCII^+^CD64^+^; dendritic cells (DC): MHCII^+^CD64^−^CD11c^+^CD103^+/−^; CFU: colony forming units; rLA: recombinant *Lactobacillus acidophilus*.

The uptake of GAD40 into Peyer’s patches and trafficking to mesenteric lymph nodes was assessed by direct delivery of GAD40 and GAD31 into an isolated section of the small intestine of BALB/cJ mice under general anesthesia using the intestinal loop method. This method removes the variable of gastrointestinal transit time and bypasses interference by gastric acid and bile [17]. Both the GAD31 and GAD40 rLA strains possess erythromycin resistance plasmids, which facilitated the selection of rLA on erythromycin-containing MRS agar plates from lysed single-cell suspensions of Peyer’s patches and mesenteric lymph nodes. Results from the intestinal loop assay showed that there was no difference in uptake of GAD31 and GAD40 into Peyer’s patches. There was robust uptake of both rLA strains in Peyer’s patches (Figure 2A). The presence of FimH did result in a significant increase in GAD40 trafficking to mesenteric lymph nodes compared to GAD31 (Figure 2B). Lactic acid bacteria have been reported to traffic to the MLN through both phagocytes and local lymphatics [21]. We hypothesized that the N-term FimH was activating antigen-presenting cells (APCs), which then increased GAD40 trafficking to the mesenteric lymph nodes.

To test this hypothesis, BALB/cJ mice were orally gavaged with CellTrace Violet-labeled GAD31 and GAD40. Mesenteric lymph nodes were collected, processed into single-cell suspensions, and analyzed by flow cytometry. Samples were analyzed for CellTrace Violet-positive (rLA) events (Supplemental Figure S4B) and association with MHCII^+^CD64^+^ macrophages and MHCII^+^CD64^−^CD11c^+^CD103^+/−^ dendritic cells (DCs) [22,23]. The results were similar to the intestinal loop and showed that GAD40 trafficked to mesenteric lymph nodes in larger numbers than GAD31 (Figure 2C). The APC analysis found that GAD40 was predominantly associated with MHCII^+^ APCs including macrophages and DCs, especially CD103^+^ DCs (Figure 2D). Very few GAD31 were detected in the mesenteric lymph nodes by flow cytometry analysis (*n* = 4), and none of these events were associated with CD103^+^ DCs (MHCII^−^, *n* = 1; MHCII^+^CD64^+^ *n* = 3). The significance of these results is unknown as the number of events was very low.

### 3.3. N-Term FimH Displayed on Surface of rLA Does Not Activate TLR4

FimH has been identified as a TLR4 ligand [10]. To determine if TLR4 binding was the mechanism of GAD40 immune activation, GAD40 and GAD31 were incubated with the HEK-Blue TLR4 reporter cell line. Control cells incubated with the TLR4 ligand LPS activated appropriately. TLR4 activation by GAD40 could not be detected (Supplemental Figure S3).

### 3.4. N-Term FimH Increases Expression of Retinaldehyde Dehydrogenase 1 and Decreases Expression of IL-21 in Peyer’s Patches and Mesenteric Lymph Nodes

**Figure 3:**
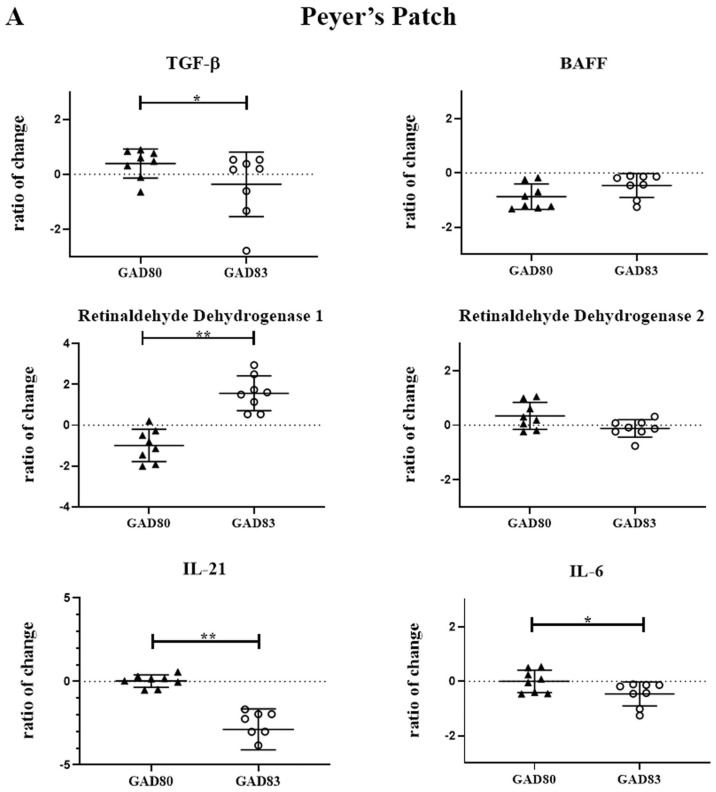

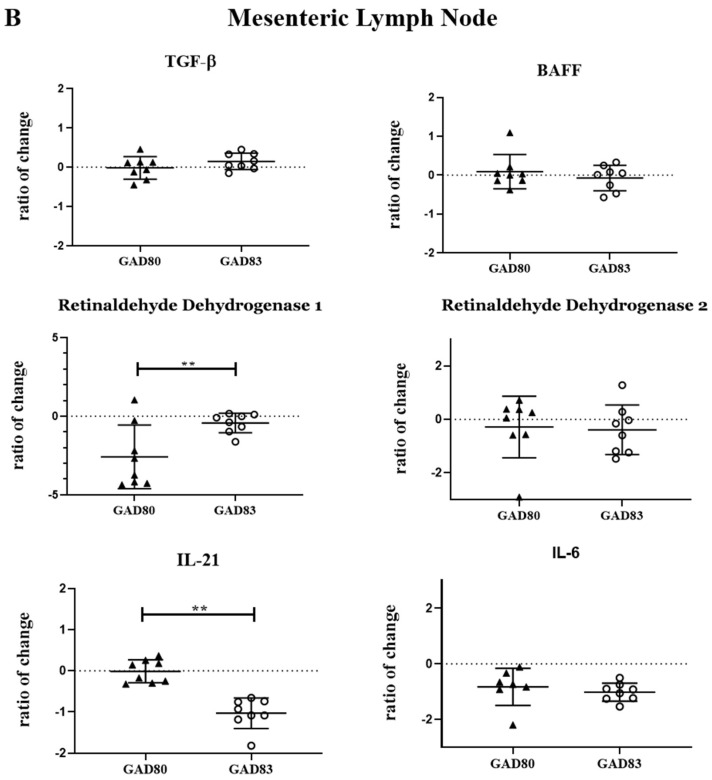
**Peyer’s patch and mesenteric lymph node cytokine and enzyme expression following oral dosing of GAD80 and GAD83.** Peyer’s patch (**A**) and mesenteric lymph node (**B**) cytokine and enzyme fold change compared to mice gavaged with dosing buffer only. * *p* < 0.1 and ** *p* < 0.05 based on a Kruskal–Wallis one-way ANOVA. Y axis is shown in Log2.

To evaluate the effect of FimH on cytokines and enzymes important for IgA class switching and secretion, we measured mRNA levels of selected cytokines and enzymes in the Peyer’s patches and mesenteric lymph nodes. Cytokines and enzymes evaluated included: TGF-β, TTNTSF13B (BAFF), ALADH1A1 (retinaldehyde dehydrogenase 1), ALADH1A2 (retinaldehyde dehydrogenase 2), IL-6, and IL-21. Transcript levels were measured 18 h after oral delivery of rLA and are reported as the fold change compared to mice gavaged with dosing buffer only (Figure 3A,B). (Ct values are shown in Supplemental Table S4).

Significant differences (*p* < 0.05) were observed in retinaldehyde dehydrogenase 1 and IL-21 in both the Peyer’s patch and mesenteric lymph nodes of the GAD83-dosed group compared to GAD80. GAD83 also had decreased TGF-β (*p* < 0.1) and IL-6 (*p* < 0.1) compared to GAD80 in the Peyer’s patch.

## 4. Discussion

Identification and development of adjuvants to induce robust mucosal and systemic immune responses is a high priority for next-generation mucosal vaccines. Oral vaccines have clear advantages over parenterally delivered vaccines including ease of delivery and the ability to induce mucosal immune responses which can protect both local (site of immune induction) and distant mucosal sites via the “common mucosal immune system” [24]. Mucosal subunit vaccines have been difficult to develop, but probiotic and lactic acid bacteria, including *Lactobacillus acidophilus,* have clear advantages as mucosal vaccine vectors due to their gastric acid and bile resistance, mucus-binding proteins, and endogenous activation of innate immune responses. Additionally, the ability to genetically modify *L. acidophilus* facilitates its development as a stable subunit vaccine vector against numerous pathogens [5,25,26,27,28,29,30,31,32,33]. The identification of immune stimulating peptide and proteins for lactic acid bacteria has been an active area of research [34]. Various bacterial proteins have been investigated as lactic acid bacteria adjuvants and have been shown to increase immune induction.

Here, we assessed expression of the N-terminal ligand-binding domain of the *E. coli* Type I fimbriae protein, FimH, by *L. acidophilus,* and we assessed its effect on immune stimulation for our rLA oral vaccine platform. To date, FimH has not been evaluated in this system. rLA was able to display the N-terminal domain of FimH (N-term FimH) on the bacterial surface, and N-term FimH maintained its biologic ability to bind to the M cell ligand GP2. Additionally, rLA surface-displaying N-term FimH had increased trafficking to mesenteric lymph nodes, increased association with dendritic cells and macrophages in the mesenteric lymph node, and induced significant alterations in the transcription of retinaldehyde dehydrogenase 1 and IL-21 in both Peyer’s patches and mesenteric lymph nodes.

Compared to previous studies using FimH to target Peyer’s patches, we did not observe differences in uptake of rLA into Peyer’s patches between strains that did and did not surface display N-term FimH [12]. Instead, we found that there was robust uptake of all strains of rLA into Peyer’s patches. This is likely because *L. acidophilus* possess an endogenous M cell-binding protein, uromodulin, and therefore N-term FimH expression did not increase rLA M cell binding and Peyer’s patch uptake [35]. It is clear from the intestinal loop uptake experiment and flow cytometry analysis that N-term FimH did significantly increase rLA trafficking to the mesenteric lymph nodes by APCs. Lactic acid bacteria have been reported to traffic to the mesenteric lymph nodes through both phagocytes and local lymphatics [21], and FimH appears to increase trafficking, particularly through its activation of MHCII^+^ cells. APCs are important for antigen uptake, processing, presentation, and priming of T and B cells, making them essential for inducing a protective immune response. There has been much interest in mucosal vaccines and adjuvants that specifically target dendritic cells since they are activators of the adaptive immune response and are important for driving isotype class switching and induction of IgA B cells [36]. The increased association of rLA surface-displaying N-term FimH with dendritic cells indicates the potential of FimH to enhance the adaptive immune responses to the LA vaccine platform.

The presence of N-term FimH on rLA significantly increased the enzyme retinaldehyde dehydrogenase 1 and decreased the cytokine IL-21 in Peyer’s patches and mesenteric lymph nodes. In this study, the cytokines TGF-β, BAFF, IL-21, and IL-6 were selected for evaluation because of their importance for B cell IgA class switching and IgA secretion [37]. N-term FimH surface-displaying rLA had decreased TGF-β and IL-21 in the Peyer’s patches and IL-21 in the mesenteric lymph node. TGF-β plays an important role in controlling inflammation by inhibiting pro-inflammatory signaling and has been shown to increase IgA isotype class switching in B cells [38,39]. *L. acidophilus* has been shown to increase TGF-β expression in mice (we also saw a modest increase in TGF-β in mice receiving the non-adjuvanted rLA) [40]. TGF-β can act in concert with IL-21 to increase IgA class switching in the Peyer’s patches [41]. IL-21 is also important for induction of IgA to the microbiota and reducing pro-inflammatory responses [42,43]. These results indicate that N-term FimH is driving a pro-inflammatory response compared to the non-adjuvanted rLA, which is a desired characteristic for an adjuvant. The increase in retinaldehyde dehydrogenase 1 by rLA surface-displaying N-term FimH suggests that the presence of FimH may be driving an increase in IgA class switching. Retinaldehyde dehydrogenase (ALADH1A1 and ALADH1A2) is primarily produced by dendritic cells and acts to convert retinal to retinoic acid (RA), which is important for the induction of the mucosal homing integrin α4β7 and CCR9 on T and B cells [44]. DCs in the Peyer’s patches express ALADH1A1 while mesenteric lymph node dendric cells express ALADH1A2 [44]. The increase in retinaldehyde dehydrogenase 1 (Aldh1a1) mRNA in both the Peyer’s patch and mesenteric lymph nodes is suspected to be secondary to dendritic cell uptake of N-term FimH-adjuvanted rLA and then trafficking to the mesenteric lymph node.

The exact mechanism of the APC–FimH interaction is unknown. Based on published mechanistic studies of FimH, we predicted an increased uptake into Peyer’s patches and/or TLR4 activation by our rLA surface-displaying N-term FimH. Interestingly, we found similar numbers of rLA in Peyer’s patches regardless of the presence of FimH and no TLR4 activation as measured using a HEK-Blue human TLR4 cell line. The lack of TLR4 activation is surprising as it conflicts with previous studies that documented TLR4 activation by FimH [10,45]. In these studies, either full-length FimH or bacteria expressing full-length FimH were used, which may explain the discrepancy with our results. Protein modeling studies evaluating the TLR4-binding domain of FimH have shown that the FimH C-terminal domain may have the strongest interaction with TLR4 and predicted the FimH N-terminal binding of TLR4 to be weak [46]. It is possible that the FimH N-terminal domain expressed on the surface of rLA is weakly activating TLR4 but at a level too low to be detected by our in vitro TLR4 assay. FimH has been shown to bind to CD48 on macrophages and enhance bacterial survival in phagosomes [11]. The majority of the FimH-adjuvanted LA in the mesenteric lymph nodes was associated with CD64^+^ macrophages, but this does not fully explain the results reported here. Further studies to identify the mechanism of immune activation by N-term FimH are needed.

## 5. Conclusions

The *E. coli* Type I fimbriae protein, FimH, can be added to the growing list of proteins that can enhance immune activation by the rLA oral vaccine platform. Further mechanistic studies and evaluation of B and T cell induction are needed to further assess the ability of FimH to serve as an rLA adjuvant.

## Figures and Tables

**Table 1:** List of plasmids and *L. acidophilus* strains.

**Plasmids**				
**Name**	**Surface-Expressed Protein**	**Tag**	**Surface Anchor**	
**pD001**	FimH	No tag	Amub	
**pD002**	FimH	5′-FLAG-tag	Amub	
**pD003**	FimH	3′-FLAG-tag	Amub	
**LA Strains**				
**Name**	**slpA Epitope Insert**	**Plasmid**	**Antibiotic Resistance**	**Reference**
**NCK56**	None	None	None	NCFM Strain
**GAD31**	HIV-1 MPER	None	Erythromycin	[5]
**GAD40**	HIV-1 MPER	pD001	Erythromycin	Here
**GAD41**	HIV-1 MPER	pD002	Erythromycin	Here
**GAD42**	HIV-1 MPER	pD003	Erythromycin	Here
**GAD80**	VP8-10pep	None	Erythromycin	Here
**GAD83**	VP8-10pep	pD001	Erythromycin	Here

## Data Availability

The flow cytometry data presented in Figure 2 and Supplemental Figure S4 are openly available at Mendeley Data, V1, doi: 10.17632/5hk4c5t236.1.

## Supplementary Materials

The following supporting information can be downloaded at: https://www.mdpi.com/article/10.3390/vaccines11071162/s1, Figure S1: Flow cytometry histography of GAD83 N-term FimH surface expression; Figure S2: N-term anti-flag uncropped Western blot; Figure S3: HEK-Blue human TLR4 activation assay; Figure S4: Flow gating strategy and rLA uptake scatter plots Table S1: FimH protein sequence, gene block sequences, and primers; Table S2: Cytokine and enzyme primer pairs and probes sequences; Table S3: Figure 1A Western blot densitometry readings; Table S4: RT-PCR cytokine and enzyme assay Ct-values.
