# Supplementary material for: Expression of E. coli FimH Enhances Trafficking of an Orally Delivered Lactobacillus acidophilus Vaccine to Immune Inductive Sites via Antigen-Presenting Cells
Source: Vaccines (Basel). 2023 Jun 27;11(7):1162. doi: 10.3390/vaccines11071162 (PMC10384470; doi:10.3390/vaccines11071162)
Supplement: Supplementary file 1 [file vaccines-11-01162-s001.zip › vaccines-2386679-supplementary.pdf]

Supplemental Table S1. FimH protein sequence, gene block sequences, and primers.

N-terminal FimH sequence		FACKTANGTAIPIGGGSANVYVNLAPVVNVGQNLVVDLSTQIF<br>CHNDYPETIDYVTLQSAYGGVLSNFSGTVKYSGSSYPFPTTSETP<br>RVVYNSRTDKPWPVALYLTPVSSAGGVAIKAGSLIAVLILRQTN<br>NYNSDDFQFVWNIYANNDVVVPTGG
FimH 5′-FLAG-tag gene block		CTGTAAAGGCAGATGAAGTTGATGATGCTAGCCGGCCGGA<br>CTACAAAGATGACGATGATAATTTGCATGCAAAACCGCTA<br>ATGGAACCGCCATTCCAATTGGTGGTGGCAGTGCAAATGTC<br>TATGTAAACTTGGCCCCAGTTGTCAATGTCGGACAAAATCT<br>TGTAGTAGATTTAAGCACTCAAATTTTTTGTCATAACGACT<br>ATCCAGAGACCATCACTGATTACGTTACCTTACAACGCGGT<br>TCAGCATACGGAGGTGTCTTATCTAACTTCTCAGGAACTGT<br>AAAGTATAGTGGTTCAAGTTACCCATTCCCAACCACTTCAG<br>AGACACCTCGTGTCGTTTACAATAGCAGAACAGATAAACC<br>TTGGCCTGTCGCCTTATACCTTACCCCTGTAAGCTCAGCAG<br>GTGGAGTCGCCATCAAAGCAGGCTCATTAATCGCTGTATTA<br>ATTTTACGCCAGACTAATAACTATAACTCTGATGACTTTCA<br>ATTCGTCTGGAATATCTATGCCAACAATGACGTAGTCGTAC<br>CAACAGGCGGCGGTACCGTGCCAACAGTTACTCCAAC
FimH 3′-FLAG-tag gene block		CTGTAAAGGCAGATGAAGTTGATGATGCTAGCCGGCCGTTT<br>GCATGTAAAACTGCCAACGGAACAGCCATCCCTATTGGTG<br>GTGGTTCTGCTAATGTTTATGTAAATTTAGCCCCTGTAGTAA<br>ATGTCGGCCAGAACTTGGTCGTAGATCTTAGTACTCAGATC<br>TTTTGTCATAACGATTATCCTGAGACCATCACTGATTATGTC<br>ACATTACAGCGAGGCTCAGCCTACGGTGGTGTTTTATCAAA<br>CTTCTCTGGAACTGTAAAGTATAGCGGAAGCTCTTATCCTTT<br>TCCAACTACCAGTGAAACTCCACGTGTAGTCTACAATAGCC<br>GAACTGATAAGCCATGGCCTGTTGCTCTTTATCTTACTCCTG<br>TAAGTAGCGCCGGTGGTGTAGCAATTAAAGCCGGAAGCTT<br>GATCGCTGTCTTGATTTTAAGACAGACAAATAACTATAATT<br>CAGACGATTTCCAGTTTGTATGGAACATCTACGCAAATAAT<br>GATGTCGTCGTCCCAACTGGCGGCGATTACAAGGACGATG<br>ATGACAAAGGTACCGTGCCAACAGTTACTCCAAC
**Primers**		
**AV-09**		ctg taa agg cag atg aag ttg atg
**AV-10**		gtt gga gta act gtt ggc agc g
**AV-29**		gcc gcc agt tgg gac gac gac at
**AV-30**		gtt gga gta act gtt ggc acg gta ccg ccg cca gtt ggg acg acg aca t
**AV-11**		ttt cgc cca gcg cta tga aaa gga tgg tgg gtt gt
**AV-12**		agc ggg ttt aaa ctc aat ggt gat ggt gat gat gtg tag tgt ggg gag tcc c

Supplemental Table S2. Cytokine and enzyme primer pairs and probe sequences.

Gene	Primer Sequences	Probe Sequence
Hprt<br>Exon: 6-7	5′-AAC AAA GTC TGG CCT GTA TCC-3′<br>5′-CCC CAA AAT GGT TAA GGT TGC-3′	5′-/56-FAM/CTT GCT GGT/ZEN/GAA AAG GAC CTC TCG GAA/3IABkFQ/-3′
B2m<br>Exon: 1-2	5′-GGG TGG AAC TGT GTT ACG TAG-3′<br>5′-TGG TCT TTC TGG TGC TTG TC-3′	5′-/56-FAM/CCG GAG AAT/ZEN/GGG AAG CCG AAC ATA C/3IABkFQ/-3′
Tgfb1<br>Exon: 1-2	5′-CCG AAT GTC TGA CGT ATT GAA GA-3′<br>5′-GCG GAC TAC TAT GCT AAA GAG G-3′	5′-/5HEX/ATA GAT GGCZEN/GTT GTT GCG GTC CA/3IABkFQ/-3′
Tnfsf13b<br>Exon: 6-7	5′-TCA TCT CCT TCT TCC AGC CT-3′<br>5′-GAC CCT GTT CCG ATG TAT TCA G-3′	5′-/56-FAM/ACA CTG CCC/ZEN/AAC AAT TCC TGC TAC T/3IABkFQ/-3′
Aldh1a1<br>Exon: 11-13	5′-ACC CAG TTC TCT TCC ATT TCC-3′<br>5′-CAT CAC TGT GTC ATC TGC TCT-3′	5′-/56-FAM/ACA CTG CCC/ZEN/AAC AAT TCC TGC TAC T/3IABkFQ/-3′
Aldh1a2<br>Exon: 8-9	5′-CAC TGG CCT TGG TTG AAG A-3′<br>5′-GAA GTA ACC TGA AGA GAG TGA CC-3′	5′-/5HEX/AGA TGC TGA/ZEN/CTT GGA CTA CGC TGT G/3IABkFQ/-3′
Il21<br>Exon: 1-3	5′-GGT TTG ATG GCT TGA GTT TGG-3′<br>5′-TGA CTT GGA TCC TGA ACT TCT ATC-3′	5′-/5HEX/TGC TCA CAG/ZEN/TGC CCC TTT ACA TCT T/3IABkFQ/-3′
Il6<br>Exon: 4-5	5′-TCC TTA GCC ACT CCT TCT GT-3′<br>5′-AGC CAG AGT CCT TCA GAG A-3′	5′-/56-FAM/AGT TAA CCC/ZEN/ACA CCA CCC CAG C/3IABkFQ/-3′

Supplemental Table S3. Figure 1A Western blot densitometry readings.

Lane	Lane Label	Band No.	Mol. Wt. (kDa)	Relative Front	Adj. Volume (Int)	Volume (Int)	Band %	Lane %
1	MW Std	1	250.00	0.100877	794808	1972782	1.85	1.84
1	MW Std	2	150.00	0.201754	1677417	3296370	3.91	3.87
1	MW Std	3	100.00	0.302632	1480710	2918139	3.45	3.42
1	MW Std	4	75.00	0.364035	4943253	6686127	11.52	11.41
1	MW Std	5	50.00	0.491228	14597415	18067770	34.02	33.71
1	MW Std	6	37.00	0.596491	8569155	10811010	19.97	19.79
1	MW Std	7	25.00	0.72807	6899256	9016014	16.08	15.93
1	MW Std	8	20.00	0.802632	2578128	3986367	6.01	5.95
1	MW Std	9	15.00	0.903509	355446	809865	0.83	0.82
1	MW Std	10	10.00	0.964912	1008567	1561959	2.35	2.33
2	GAD31	None						
3	GAD41	1	48.76	0.5	561855	848085	100.00	18.01
4	GAD42	1	100.00	0.302632	2277156	4200252	65.00	35.90
4	GAD42	2	48.15	0.504386	1226022	1678740	35.00	19.33

Supplemental Table S4. RT-PCR cytokine and enzyme assay Ct-values.

Peyer's Patch	Ct								
Group	Mouse	Hprt	B2M	TGF-β	aldh1a1	aldh1a2	BAFF	IL-21	IL-6
GAD80	1	24.59	20.59	23.66	25.99	32.64	31.62	29.42	33.41
	2	24.37	19.98	23.54	24.62	32.51	31.24	28.94	33.41
	3	24.49	20.31	23.24	26.35	31.51	31.35	28.63	32.65
	4	26.46	23.16	27.13	27.03	33.86	33.25	32.09	35.35
	5	24.51	20.19	23.13	26.22	32.15	31.33	28.97	32.63
	6	25.69	22.07	25.67	25.62	33.38	31.80	30.51	35.13
	7	25.03	21.49	24.17	26.02	33.54	31.85	30.57	34.44
	8	25.43	22.23	25.03	26.92	33.74	31.82	30.33	34.53
GAD83	1	26.24	22.78	26.12	25.86	32.84	32.21	32.94	35.29
	2	25.14	20.34	23.88	24.15	33.07	30.82	31.49	33.65
	3	25.58	21.17	24.85	24.17	33.15	31.01	32.41	34.34
	4	26.31	22.10	27.21	23.21	34.43	30.64	34.02	NA
	6	26.27	21.28	28.24	23.23	34.63	30.26	35.90	35.57
	7	26.12	21.30	25.99	23.92	33.72	30.19	33.51	NA
	8	25.94	21.23	25.09	23.92	33.60	30.57	34.21	34.82
	9	25.30	20.91	24.40	23.55	33.29	31.11	31.58	34.00
Mesenteric Lymph Node	Ct								
Group	Mouse	Hprt	B2M	TGF-β	aldh1a1	aldh1a2	BAFF	IL-21	IL-6
GAD80	1	24.66	19.28	23.22	31.82	30.18	32.82	31.94	34.20
	2	24.61	19.34	23.65	35.62	29.45	32.51	30.13	33.71
	3	24.53	18.88	23.58	35.25	28.97	32.40	30.33	33.63
	4	24.83	19.56	23.81	35.32	29.34	33.07	30.24	33.32
	5	25.99	21.44	25.72	35.77	31.81	33.37	31.65	35.55
	6	24.30	18.83	22.99	29.89	28.37	32.45	29.69	NA
	7	23.92	18.41	22.26	32.73	31.60	32.29	29.76	34.37
	8	24.20	19.01	23.04	31.25	29.72	32.35	30.16	32.94
GAD83	1	24.86	19.40	23.35	33.12	28.37	32.54	31.09	34.19
	2	24.61	19.03	23.34	32.16	30.54	32.82	31.20	34.13
	3	24.45	19.10	23.35	31.15	29.45	32.46	31.16	34.01
	4	24.48	19.41	23.45	31.98	29.19	32.61	31.00	33.87
	6	24.73	19.06	23.29	31.36	30.02	32.39	30.94	33.64
	7	24.89	19.24	23.17	31.27	31.07	33.38	31.55	34.61
	8	25.08	19.61	23.55	32.10	31.11	33.56	32.46	34.25
	9	24.81	19.37	23.79	31.35	29.65	32.82	31.32	33.60

Supplemental Figure S1. Flow cytometry histography of GAD83 N-term FimH surface expression.

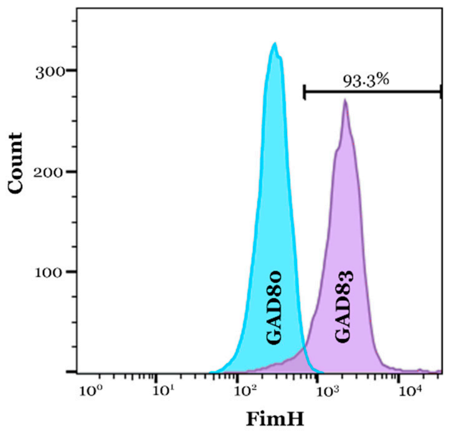

Supplemental Figure S2. N-term FimH anti-flag uncropped Western blot. Lanes: 1: Bio-Rad Precision Plus Protein WesternC Blotting Standard 2: GAD31 3: GAD41 4: GAD42 5: Blank 6: Bio-Rad Precision Plus Protein WesternC Blotting Standard 7: GAD31 8: GAD41 9: GAD42

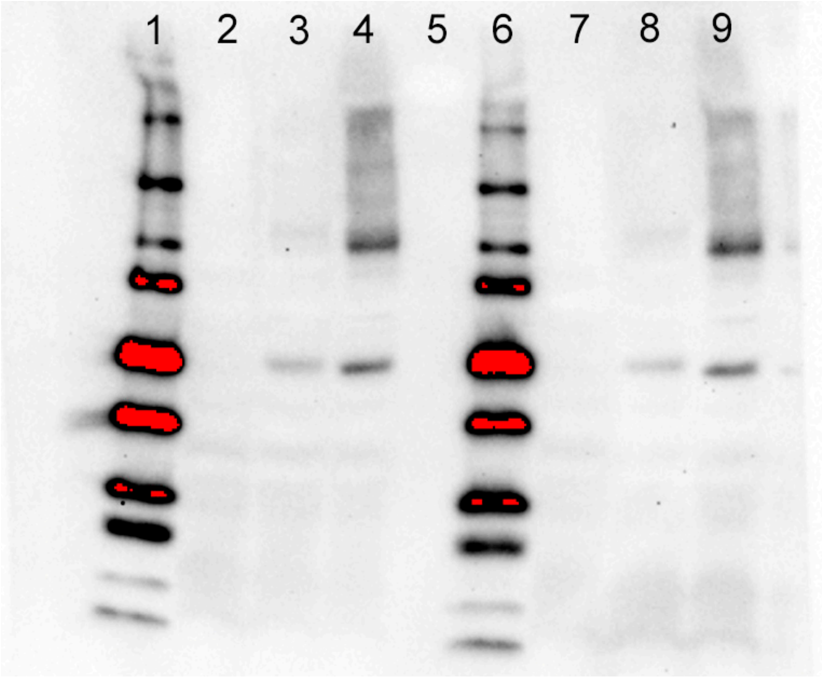

Supplemental Figure S3. HEK-Blue human TLR4 activation assay. HEK-Blue human TLR4 and Null2 plated cells were stimulated overnight with PBS, LPS (10 ng, 100 ng), GAD31 (25 MOI, 50 MOI), or GAD40 expressing the N-terminal domain of FimH (25 MOI, 50 MOI). Absorbance was read at 650 nm and relative increase in HEK-Blue TLR4 cell NF-kB-induced secreted alkaline phosphatase (SEAP) versus HEK-Blue Null2 cells was determined. GAD40 did not increase SEAP secretion when compared to GAD31 or PBS controls.

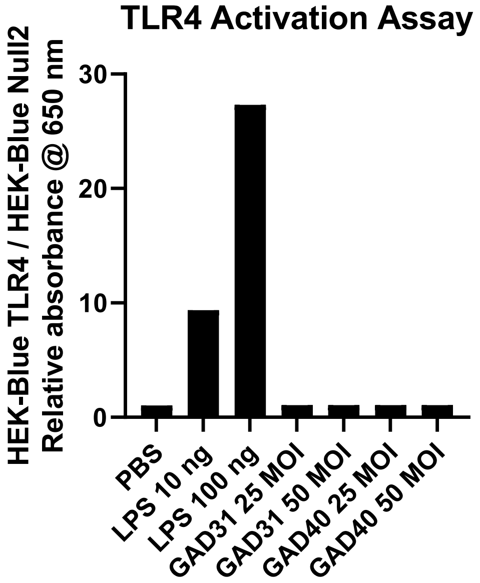

Supplemental Figure S4. Flow gating strategy and rLA uptake scatter plots. (A) Antigen presenting cell gating strategy. All gates were set using fluorescence minus one (FMO) controls. (B) GAD31 and (C) GAD40 scatter plots of mesenteric lymph nodes from BALB/cJ mice orally gavaged with CellTrace Violet labeled LA. Scatter plots of representative of 1 of 5 sample tubes run per mouse.

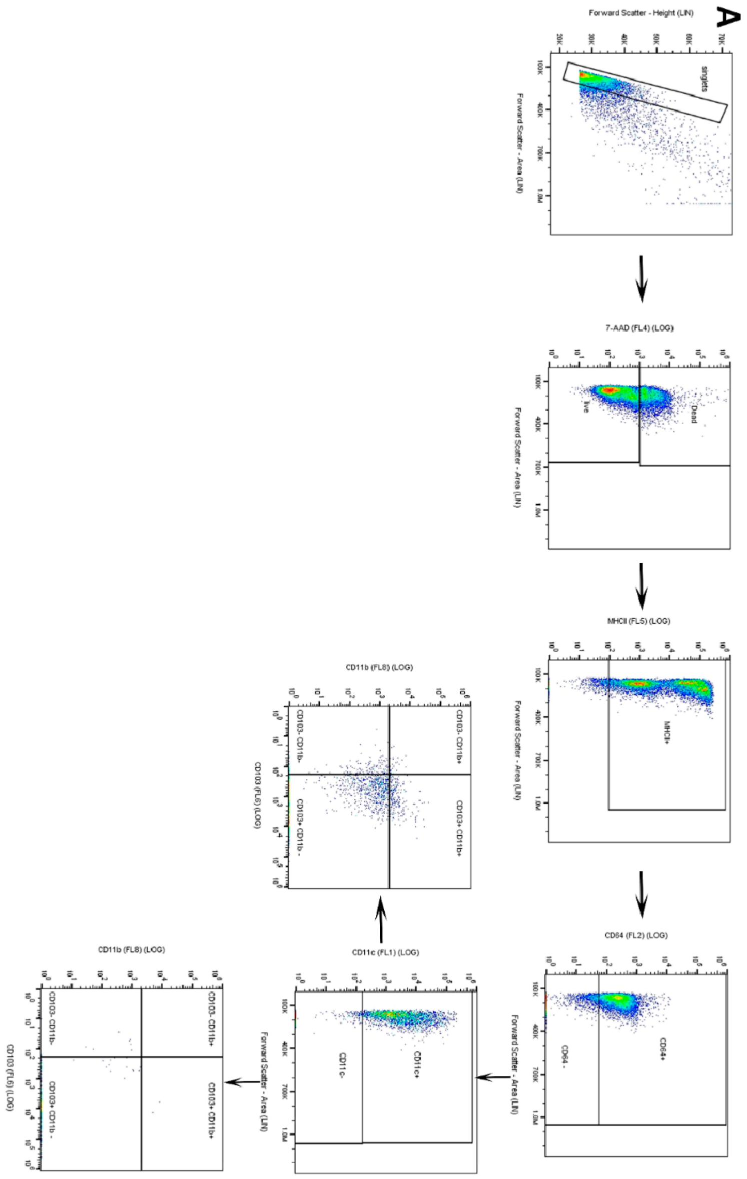

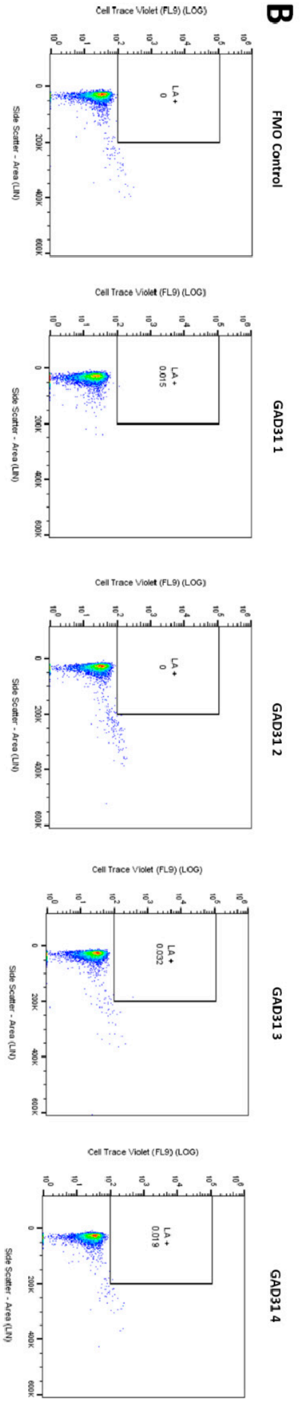

B
FMO Control
GAD31 1
GAD31 2
GAD31 3
GAD31 4
LA +
0
0.015
0
0.032
0.019
Cell Trace Violet (FL9) (LOG)
Side Scatter - Area (LIN)
200K
400K
600K

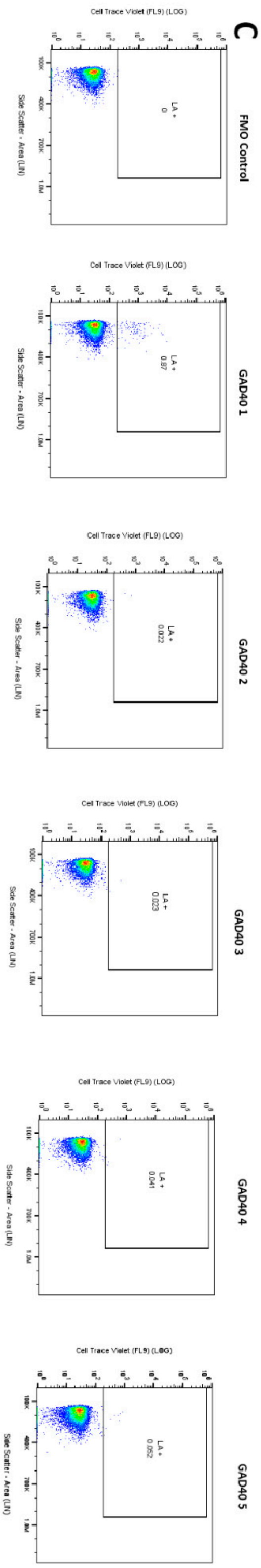
C
FMO Control
GAD40 1
GAD40 2
GAD40 3
GAD40 4
GAD40 5
Cell Trace Violet (FL9) (LOG)
Side Scatter - Area (LIN)
LA +
0
0.87
0.022
0.023
0.041
0.052
